# MgCl_2_-Supported Titanium Ziegler-Natta Catalyst Using Carbon Dioxide-Based Poly(propylene ether carbonate) Diols as Internal Electron Donor for 1-Butene Polymerization

**DOI:** 10.3390/polym9110627

**Published:** 2017-11-17

**Authors:** Xiaopeng Cui, Qing Bai, Kai Ma, Min Yang, Binyuan Liu

**Affiliations:** Department of Polymer Science and Engineering, Hebei University of Technology, Tianjin 300130, China; xpcuihebut@163.com (X.C.); baiq1991@163.com (Q.B.); gwwymk123@163.com (K.M.); polyym@163.com (M.Y.)

**Keywords:** poly(propylene ether carbonate) diols, 1-butene polymerization, internal electron donor, Ziegler-Natta catalyst

## Abstract

MgCl_2_-supported titanium Ziegler-Natta catalyst containing CO_2_-based poly(propylene ether carbonate) diols as a potential internal electron donor (IED) was synthesized and employed for 1-butene polymerization. When compared with the Ziegler-Natta catalyst using poly(polypropylene glycol) as IED, the catalyst prepared with poly(propylene ether carbonate) diols showed good particle morphology, higher activity and stereoselectivity. The results suggested that existence of the carbonate group within the structure of poly(propylene ether carbonate) diols truly plays an important role in improving the performance of the catalyst for the 1-butene polymerization.

## 1. Introduction

Isotactic poly(1-butene) (PB) is one of the polyolefin materials with outstanding physical and mechanical properties, such as high-temperature creep resistance, environmental stress cracking resistance, excellent elastic recovery, and good resistance to chemical corrosion. Owing to its excellent comprehensive properties, the isotactic PB is widely used in the preparation of pipes, thin films, and various containers [1]. At the same time, the preparation of PB materials is one of the important ways to balance the problem of the excess 1-butene resources [2]. At present, the catalyst used in PB industry is still the traditional heterogeneous Ziegler-Natta catalyst that is assisted by alkylaluminium, especially the fourth-generation Ziegler-Natta catalyst, which is prepared by embedding TiCl_4_ on MgCl_2_ support with diisobutylphthalate (DIBP) as potential internal electron donor (IED) [3]. It is well known that IEDs play an important role in tuning the performance of catalysts and the properties of corresponding polymers [4,5,6], including the activity, isotacticity index, molecular weight (MW), molecular weight distribution (MWD) [7,8,9], and hydrogen response of the catalysts [10]. Therefore, it is of great significance to explore potential IED with novel structure and functions for MgCl_2_-supported titanium Ziegler-Natta catalyst.

Ether compounds have been widely used as IED in MgCl_2_-supported titanium Ziegler-Natta catalyst system. The 1, 3-diether-based IED made Ziegler-Natta catalyst possibly to obtain polymers with high isotacticity and narrow MWD without using an external donor (ED) during the polymerization process [11]. Recently, Sami et al. [12] examined the poly(ethylene glycol) (PEG) and poly(tetrahydrofuran) (PTHF) containing a certain number of ether bonds as IED to prepare novel Ziegler-Natta catalyst. It is interesting to find that catalysts that were prepared with these polyether IEDs showed good activity and comonomer response in ethylene polymerizations. Basell company and Dow global technology company reported a compound containing both ether and ester bonds as IED [13,14]. The catalysts prepared with this kind of IED showed good activity and stereoselectivity in propylene polymerization, but the synthesis cost of these compounds is high to some extent. Chen et al. [15] have reported that di(propylene glycol) dibenzoate and tri(propylene glycol) dibenzoate can also be used as IED and the activity of the catalysts consisting of these IEDs were higher than that of the catalyst with diisobutylphthalate (DIBP) as IED in propylene polymerization. Meanwhile, thanks to its bifunctional molecular structure containing both multi-ether segment and ester bond, the MWD of polypropylene that was obtained by these catalysts were broad up to 9.5. To our knowledge, there is only one study related to carbonic ester in the Ziegler-Natta catalyst, in which it was used as ED in the propylene polymerization process to achieve a smooth polymerization [16]. However, no report concerns the carbonate compound as IED in preparation of MgCl_2_-supported Ziegler-Natta catalyst.

According to the above-mentioned analyses, in this study we utilize poly(propylene ether carbonate) diols (PPEC) as IED in MgCl_2_-supported titanium Ziegler-Natta catalyst system and applied it to 1-butene polymerization. This kind of long chain compound contains carbonate group and polyether segment is relatively harmless when compared with the commonly used DIBP in the industry. Importantly, PPEC can be synthesized by copolymerization of CO_2_ and epoxides in the presence of transfer reagent under double metal cyanide complex, which can not only make full use of greenhouse-gas CO_2_, but also reduce the energy consumption as compared to polyether polyols analogues in the production process [17]. Herein, two aspects were paid more attention, one focuses upon the influence of IED on the physical properties of the catalyst, the other is paid to the effect of the carbonate group in IED on the active centers of the corresponding catalyst. At last, the catalytic behaviors in the 1-butene polymerization were investigated.

## 2. Materials and Methods

### 2.1. Materials

**Figure 1:**
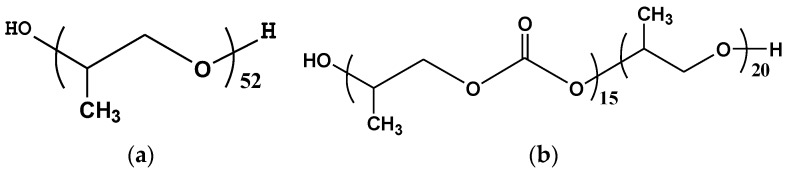
Structures of IED-1 (**a**) and IED-2 (**b**).

All of the manipulations of the moisture or air sensitive materials were carried out under a dry argon atmosphere. Toluene and *n*-hexane were dried over 4 Å molecular sieves for 48 h. Cyclohexylmethyldimethoxysilane (CHMMS) was dried over 4 Å molecular sieve and was stored under argon atmosphere. The magnesium chloride ethanol support (MgCl_2_·2.5C_2_H_5_OH) was provided by Daqing chemical research center (Daqing, China). Triethylaluminum (TEA, 1 mol/L in *n*-hexane) was purchased from Yanfeng Science and Technology Company (Beijing, China) and was used as received. Poly(polypropylene glycol) (PPG) (Figure 1a, PPG-3000, IED-1, *M*_n_ = 3000 g/mol, hydroxyl number is 41.3 mg KOH/g) was purchased from Haian petroleum and chemical company (Nantong, China). PPEC (Figure 1b, IED-2) was prepared according to the modified literatures [18,19] and was dried on a vacuum at 110 °C for 6 h, whose *M*_n_ = 2669 g/mol, hydroxyl number is 42.2 mg KOH/g, the ether content is 57 mol %. All of the other chemicals were purchased from J & K Chemical (Beijing, China) and used as received.

### 2.2. Preparation of Catalysts and Metal Complexes

TiCl_4_ (190 mL) was added into five-neck flask and was stirred with a mechanical stirred. After the TiCl_4_ was cooled to −15 °C, MgCl_2_·2.5C_2_H_5_OH (7.8 g) was added into the system and then heated to 90 °C for 1 h. Then, a certain amount of IED was added and stirred for 30 min. The system maintained at 110 °C for 2.5 h. Finally, the resulting precipitate was washed with toluene and *n*–hexane and then dried under vacuum at 60 °C. The catalyst without IED was designated as Cat-1. The catalysts bearing IED-1 and IED-2 were designated as Cat-2, Cat-3, respectively. MgCl_2_·IED samples were prepared by interaction of a suspension of MgCl_2_ (1.50 g, 12.6 mmol) in toluene with corresponding excess IED at 110 °C for 6 h. The white precipitates were triply washed with toluene and *n*-hexane and were dried in vacuum. For the synthesis of TiCl_4_·IED complexes, TiCl_4_ (0.5 mL, 4.6 mmol) was added dropwise to a solution of IED (0.50 g) in toluene (20 mL). The mixture was further stirred at 110 °C for 6 h. Then, the solvent was removed by filtration, and the residue was washed with toluene and *n*-hexane three times and dried in vacuum.

### 2.3. 1-Butene Polymerization

1-Butene slurry polymerization was conducted in a 250 mL three-neck flask equipped with a thermostatic system and a magnetic stirrer. The reactor was evacuated and purged with argon and 1-butene. *N*-hexane (50 mL), CHMMS, H_2_, desired amounts of AlEt_3_ and 10 mg of catalyst were filled into the reactor. Stirring the mixture for 2 h at 30 °C. The reaction was quenched by adding 5% HCl/ethanol solution. The precipitates were filtrated and washed by ethanol three times. Then, the resulting polymer was collected and dried in vacuum at 45 °C to constant weight.

1-Butene bulk polymerization was conducted in a 2 L stainless steel reactor equipped with a mechanical seal stirrer. Calculated volume of H_2_ was introduced to the reactor. Anhydrous *n*-hexane (10 mL), AlEt_3_, and CHMMS were injected into the feed tank by syringe under nitrogen. Then, 200 g liquid 1-butene monomer was introduced to the reactor along with the catalyst slurry. The reactor temperature was warmed up to 30 °C during 10 min and polymerization was maintained at the temperature for 2 h. After that, the mixture was poured into acidified ethanol solution. The crude PB was washed with ethanol three times. After it, the PB was dried in vacuum at 45 °C to constant weight.

### 2.4. Characterization

Magnesium and chloride content in the catalyst were determined by titration with ethylenediaminetetraacetic acid and silver nitrate, respectively. Titanium content was determined by spectrophotometer at 410 nm in the solution of the catalyst, which was treated with sulfuric acid (7.2 mol/L) on UV-CARY300 spectrometer (Agilent Technologies, Palo Alto, CA, USA). The morphology and surface information of the support and catalysts were measured by SEM (Bern, Switzerland) on Nova Nano SEM-450 (FEI, Hillsboro, OR, USA). Brunauer-Emmett-Teller (BET) analysis was performed on ASAP-2000 (US Marks Company, Atlanta, GA, USA). The porosity and surface areas of the support and catalysts were determined by the BJH (Barrett-Joyner-Halenda) and BET (Brunauer-Emmett-Teller) methods, respectively. The particle size distribution (PSD) of the support and catalysts was measured by Matersizer 2000 (Malvern Instruments Ltd., Malvin, UK). Molecular weight (MW) and molecular weight distribution (MWD) of PB were measured by gel permeation chromatography (GPC) using a Waters Alliance GPC 2000 instrument (Waters, Milford, MA, USA) equipped with a refractive index (RI) detector and a set of u-Styragel HT columns of 106, 105, 104 and 103 pore size in series. IR spectra of catalyst and metal complexes were measured on a Bruker Vector 22 spectrometer (Bruker Optics, Karlsruhe, Germany) using Nujol mull, all spectra were recorded with a nominal resolution of 4 cm^−1^. X-ray photoelectron spectroscopy (XPS) measurements were performed with a K-Aepna (Thermo Fisher Scientific, Waltham, MA, USA) at room temperature, using monochromated Mg Kα radiation (0–1100 eV). All of the samples were prepared in a glove box and transferred under nitrogen atmosphere to prevent exposure with air. Spectra were recorded at 1 × 10^6^ Pa and the accurate binding energy (BE) of the Ti_2p3/2_ peak was determined by referencing to the Au_4f7/2_ peak at 84.0 eV. FWHM was full-width at half maximum intensity. Activity was determined in terms on the produced PB (kg) per the used Ti in the polymerization. The isotacticity index (I.I) of PB was determined as a percent insoluble in boiling diethyl ether.

## 3. Results and Discussion

### 3.1. Components and Structures of the Support and Catalysts

**Figure 2:**
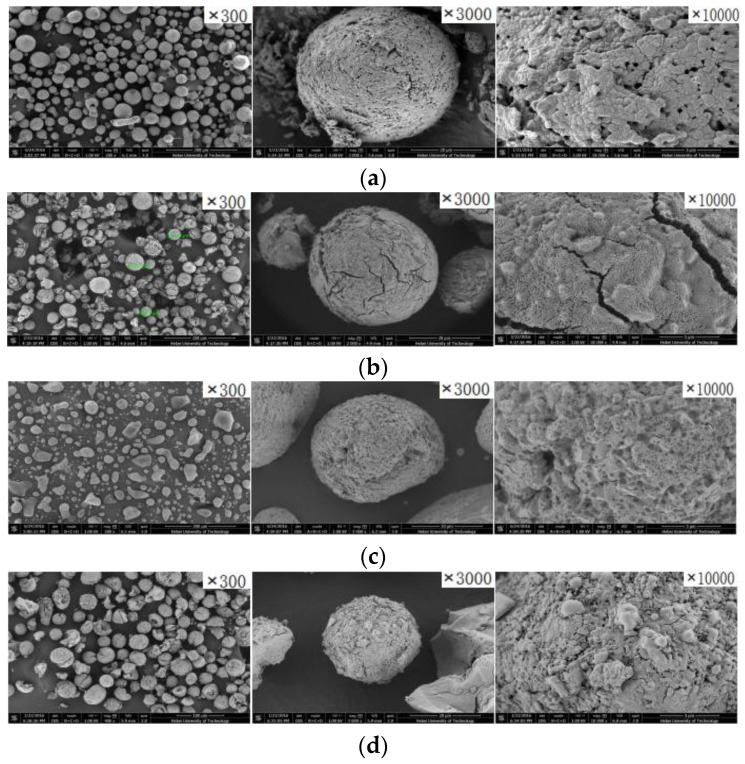
SEM images of support (**a**), Cat-1 (**b**), Cat-2 (**c**) and Cat-3 (**d**).

The morphology of the support and catalysts with different donors were presented in Figure 2. It was found that Cat-2 bearing PPG-3000 internal electron donor (IED-1) possessed fine powder and cracked particles (Figure 2c). On the contrary, Cat-3 bearing PPEC electron donor (IED-2) showed good particle morphology (Figure 2d) and less fragment. When compared with the support (Table 1), the specific surface area of the three catalysts was increased by about 20 times and the pore volume of catalysts is improved by two times after titanium loading. Additionally, the surface of the Cat-2 is found to bear less holes on the surface, which reasonably prevents the internal active centers on the catalyst to contact with the monomer and thus affect the activity for the following 1-butene polymerization.

As shown in Table 1, the Ti content of Cat-2 is higher than that of Cat-1 and Cat-3. This is attributed to the formation of some insoluble polyether/TiCl_4_ complex, it cannot be removed during the washing process [12]. Another possible reason arises from the higher interactions between the PPG with TiCl_4_ compound due to the much repeat ether group coordinating to Ti center, which further makes the excess Ti difficulty to remove during the washing process of the catalyst samples. The less Ti content in Cat-3 as compared to Cat-2 is mostly likely due to the weaker coordination of carbonate group to Ti^4+^ ion in comparison with ether group. The coordination interactions between the ether group and carbonate group with Ti ion was evidenced by IR measurement. The formation insoluble PPEC/TiCl_4_ complexes also contributed to its high Ti content like PPG electron donor performance.

### 3.2. The Particle Size Distribution of the Support and Catalysts

**Figure 3:**
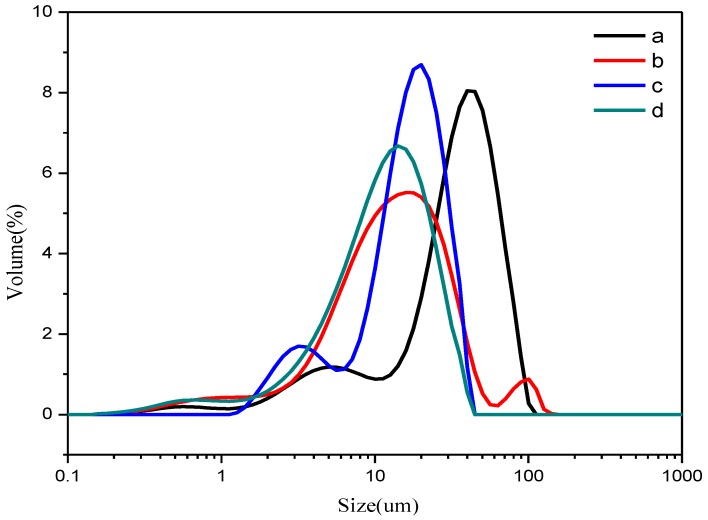
The particle size distribution of the support (a) and catalysts (Cat-1 b, Cat-2 c, Cat-3 d).

The particle size and its distribution are very important aspects of Ziegler-Natta catalysts as it affects the properties of the final polymer [20,21,22]. The previous work demonstrated that IED is an important factor to affect the particle size and its distribution of catalyst [23]. Therefore, the prepared catalysts were determined by the PSD methods. As shown in Figure 3, the small peak around 100 µm (Figure 3b) in Cat-1 without IED indicate that there is a small aggregation of catalyst particles. Cat-2 with PPG-3000 as IED, a small peak around 7 µm was observed (Figure 3c), while Cat-3 bearing PPEC (IED-2) shows a standard normal distribution. More importantly, the particle size of the three catalysts moves to small size direction in comparison with the support. It is reasonably attributed to that the removing of ethanols from the support during Ti loading make the MgCl_2_ framework of the support collapses and shrinks, resulting in a smaller average particle size. From the above observations, the existence of carbonate units in the long chain IED-2 play an essential role in controlling the catalyst particle morphology.

### 3.3. XPS and Fourier Transform Infrared Spectroscopy (FT-IR) Analyses of the Catalyst

**Figure 4:**
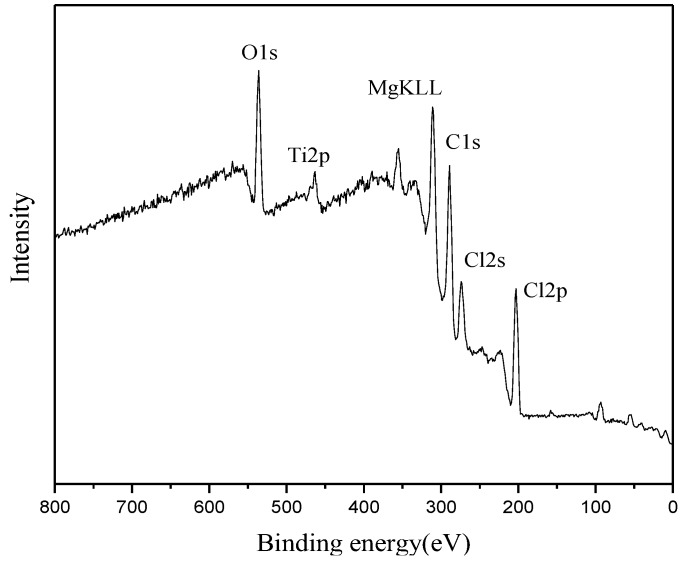
X-ray photoelectron spectroscopy (XPS) survey spectrum of Cat-3.

**Figure 5:**
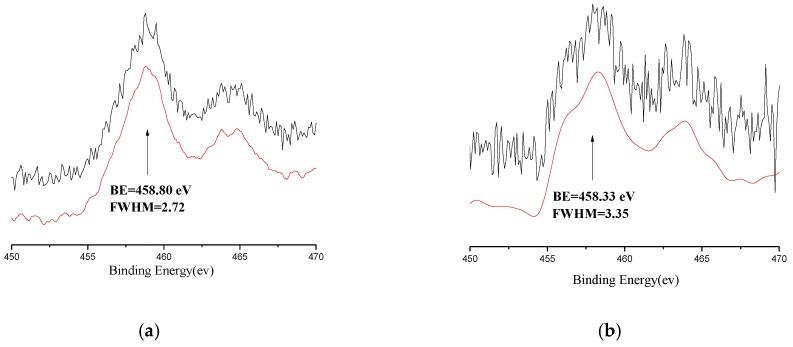
XPS spectra of Ti_2P3/2_ region of Cat-1 (**a**) and Cat-3 (**b**).

The characterization of XPS can provide important information on the chemical composition and the electronic structure of the solid sample surface [24]. Herein, in order to clarify the influence of PPEC on the oxidation state of the titanium species and the relationship between binding energy (BE) with the performance of catalyst, two types of MgCl_2_-supported titanium Ziegler-Natta catalysts containing IED-2 (Cat-3) and IED-free (Cat-1) were selected as samples. A representative survey spectrum of Cat-3 is shown in Figure 4. All of the constituent atoms of the catalyst (Ti, Mg, Cl, O and C) were observed to exist in the XPS measurable sampling depth (approximately 2 nm). Figure 5 shows the high resolution mode scan of the Ti_2p_ region for Cat-1 and Cat-3. As can be seen, the Ti_2p_ region presents a doublet center at 458.3 and 463.9 eV (Figure 5b) owing to the 2p_3/2_ and 2p_1/2_ photoelectrons from titaniums in its molecular solid-state [25,26,27,28]. The BE for Ti_2p3/2_ of Cat-1 without IED is 458.8 eV. After adding IED-2 to Cat-3, the BE of Ti_2p3/2_ shift to a lower energy region (458.3 eV) and the Ti_2p3/2_ FWHM of Cat-3 (3.35) appeared slightly broader than that of Cat-1 without IED (2.72), which suggests that Ti atom in Cat-3 lies in a more electron rich environment. This could be accounted for the higher activity and stereospecificity for Cat-3, as indicated by its Ti atom in Cat-3 with lower BE. A correlation between BE and catalyst activity, stereospecific was already reported in the previous studies [17,29], where they found that Ti atom in the supported catalyst with lower BE showed higher activity and stereospecificity. It is a truth in light of the following results in 1-butene polymerization (Cat-1 and Cat-3 in Table 2).

**Figure 6:**
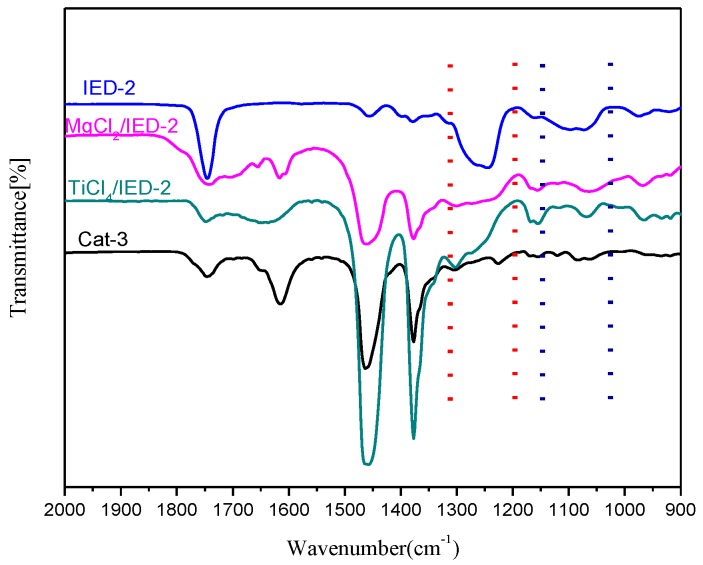
Stretching Vibration of Carbonate Group and Ether Bond of IED in TiCl_4_·IED-2, MgCl_2_·IED-2 and Cat-3 (Nujol mull sample).

To further understand how PPEC (IED-2) affects the active centers of the catalyst, interaction between the IED-2 with Mg and Ti species was studied by IR spectroscopy. As shown in Figure 6, MgCl_2_·IED-2 complex, TiCl_4_·IED-2 adduct, and Cat-3 show new absorption peak in the range of 1550–1690 cm^−1^, particularly, MgCl_2_·IED-2 complex show much complicated peak in this region. The bond characteristic of *v* (C=O) in MgCl_2_·IED-2 complex, TiCl_4_·IED-2 adduct, and Cat-3 at 1740 cm^−1^ is close to the *v* (C=O) of neat IED-2, however, the C–O bond in carbonate group occurred obvious changes for MgCl_2_·IED-2 complex, TiCl_4_·IED-2 adduct, and Cat-3, indicating that oxygen in the carbonate group coordinate to the metal via C–O bond. Furthermore, the shift of the *v* (C–O–C) bands (ether bonds) maximum of IED-2 from 1097 and 1072 cm^−1^ to a lower wavenumber at 1084 and 1063 cm^−1^, as observed for the corresponding Ziegler-Natta catalyst (Cat-3), indicates that the bonding of IED-2 to Mg and/or Ti atoms through ether oxygen atom. Therefore, the double coordination of carbonyl and ether bonds in IED-2 leads to stronger coordination between the IED-2 and the Ti atom, and provides a more electron rich environment to the titanium species on the Cat-3, which makes the IED-2 hard to be extracted by CHMMS in the process of 1-butene polymerization. This will lead to higher stereospecific properties for Cat-3 bearing IED-2.

### 3.4. Effects of Different IEDs on Activity, Isotacticity Index, Molecular Weight and Molecular Weight Distribution of PB

As shown in Table 2, Cat-3 bearing IED-2 had a higher catalytic activity than those of Cat-1 and Cat-2. The possible reason indicated by the above analyses on the surface and morphology. Besides, in comparison with Cat-1 with no IED and Cat-2 with PPG-3000 as IED, the Cat-3 bearing PPEC gives an apparent increase of PB isotacticity index (83.7% for Cat-1, 84.6% for Cat-2, and 91.7% for Cat-3). On the basis of polymerization results combined with XPS and IR study, it is considered that the difference in the polymerization behavior between the two catalysts (Cat-2 and Cat-3) is considered to stem from the coordination ability of the two different IEDs. PPEC (IED-2) has a double coordination with the catalyst via C–O bond in carbonate group and ether bonds, and its coordination ability is stronger than that of PPG (IED-1), whose coordination form is only coordinated by the ether bond with the catalyst. The double coordination provides a more electron rich environment to the titanium species (XPS results, BE = 458.3 eV) on the catalyst and improves the effect of stereotactic for titanium active centers. Thus, when compared with Cat-2, the PB prepared by Cat-3 showed the highest I.I value and Cat-3 shows a higher polymerization activity.

### 3.5. Effect of Al/Ti, Al/Si Molar Ratios and the Amount of H_2_ on the Performance of the Cat-3 in 1-Butene Polymerization

The cocatalyst triethylaluminium (TEA) plays a critical role in Ziegler-Natta catalyst system, which can initiate the catalyst and the concentration of cocatalyst can affect the valence of Ti in the active centers and then affect the performance of the catalyst [30]. So, the effect of Al/Ti molar ratio was studied in the absence of ED (Table 3, runs 1, 2, 3, 4 and 5). The results demonstrate that the activity of the catalyst increases to 9.75 (Kg PB/g Ti) firstly and then decreases with the Al/Ti molar ratios increased from 100 to 300, however, it is not obvious changes of PB isotacticity. This possibly lies in that the Ti^4+^ species can reduce to the active Ti^3+^ in the appropriate TEA, in this case, the catalytic activity increased with the increase of Al/Ti, owing to more active centers are formed. However, the excess of TEA can further reduce the active Ti^3+^ to inactive Ti^2+^, therefore further enhancing Al/Ti molar ratio results in the decrease of the activity [31,32].

Runs 6, 7, 8 and 9 show the effect of the amount of ED (CHMMS) on the polymerization. When compared with run 1, 2, 3, 4 and 5 without ED, the addition of CHMMS during the polymerization process greatly increased the isotacticity index of PB from 76.4% to 90.3%. Generally, the ED could decrease both the concentration of isotactic and atactic active centers, but atactic active centers decreased much more [30,33]. As a result, it reduced the catalyst activity but effectively increased the isotacticity index of PB.

Hydrogen (H_2_) is the most commonly used chain transfer agent in olefin polymerization, which is an effective additive to regulate the molecular weight of polymers. In addition, the H_2_ also plays important roles in the polymerization rate and I.I content [34,35]. As shown in Table 3 (run 10, 11, 12 and 13), the activity of the catalyst increased firstly and then decreased with the loading H_2_ increase. The literatures [35,36,37] have demonstrated that a few H_2_ could reactivate the less active Ti–CH(CH_2_)_2_CH_3_– species, a product from an irregular (2,1-) insertion manner, which trigger further polymerization. Besides, when the Cat-3 is employed to 1-butene bulk polymerization, the catalyst activity is enhanced by more than ten times, and I.I of PB increase from 88.7% for slurry polymerization to 94.3% (run 14 vs. run 8, Table 3).

## 4. Conclusions

Poly(propylene ether carbonate) diols was used as IED to investigate 1-butene polymerization based on heterogeneous MgCl_2_-supproted Ziegler-Natta catalyst system. The SEM, BET, PSD, and XPS results showed that the addition of poly(propylene ether carbonate) diols can effectively improve the particle morphology of the catalyst, and it also effectively affects the oxidation state and the environment of the titanium active centers through the coordination between carbonate group, ether bonds with the metal elements in the catalyst. The catalyst with poly(propylene ether carbonate) diols as IED showed a higher activity and stereospecificity.

## Figures and Tables

**Table 1:** Element content and pore structure parameters of the support and catalysts.

Catalyst	Mg content (%)	Cl content (%)	Ti content (%)	Surface area (m^2^/g)	Pore volume (cm^3^/g)	Pore size (nm)
support	10.40	33.43	-	20.80	0.12	19.43
Cat-1	15.70	69.26	7.78	467.13	0.28	3.29
Cat-2	14.01	63.40	10.78	362.30	0.33	3.98
Cat-3	15.50	63.11	6.92	364.53	0.34	3.73

**Table 2:** The effect of IED on 1-butene slurry polymerization ^a^.

Catalyst	Activity (Kg PB/g Ti)	I.I (%)	M_w_^b^ × 10^4^ (g/mol)	MWD ^b^
Cat-1	8.30	83.70	26.40	8.26
Cat-2	5.98	84.60	33.75	8.59
Cat-3	9.46	91.70	29.94	6.04

**^a^** Polymerization conditions: *n* (Al)/*n* (Ti) = 200; *n* (Al)/*n* (CHMMS) = 30; H_2_ = 4 mL; V (*n*-hexane) = 50 mL; *m* (catalyst) = 10 mg; T = 30 °C; 0.10 MPa of 1-butene pressure; t = 2 h. ^b^ GPC results.

**Table 3:** The effect of the Al/Ti, Al/Si molar ratios and H_2_ on the Cat-3 in 1-butene slurry polymerization.

Run	*n* (Al)/*n* (Ti) (mol.mol^−1^)	*n* (Al)/*n* (Si) (mol.mol^−1^)	H_2_ (mL)	Activity (Kg PB/gTi)	I.I (%)
1 ^a^	100	0	0	7.23	73.2
2 ^a^	150	0	0	8.21	74.0
3 ^a^	200	0	0	9.75	76.2
4 ^a^	250	0	0	7.71	76.4
5 ^a^	300	0	0	6.59	79.5
6 ^a^	200	40	0	4.27	90.3
7 ^a^	200	35	0	4.74	89.9
8 ^a^	200	30	0	5.55	88.7
9 ^a^	200	20	0	5.27	90.2
10 ^a^	200	30	1	6.88	89.0
11 ^a^	200	30	3	9.44	89.9
12 ^a^	200	30	4	9.46	91.7
13 ^a^	200	30	5	5.65	92.1
14 ^b^	200	30	0	59.8	94.3

^a^ 1-butene slurry polymerization conditions: V (*n*–hexane) = 50 mL; *m* (catalyst) = 10 mg; T = 30 °C; t = 2 h. ^b^ 1-butene bulk polymerization conditions: *m* (catalyst) = 20 mg; T = 30 °C; t = 2 h; *m* (1-butene) = 200 g.

## References

[1] Luciani L., Seppälä J., Löfgren B. (1988). Poly-1-butene: Its preparation, properties and challenges. Prog. Polym. Sci..

[2] Liu X.H., Liu C.G., Zhao Z.C., Huang B.C. (2010). Research progress in 1-butene copolymerization. China Synth. Rubber Ind..

[3] Li P.Y., Tu S.T., Xu T., Fu Z.S., Fan Z.Q. (2015). The influence of combined external donor and combined cocatalyst on propylene polymerization with a MgCl_2_-supported Ziegler-Natta catalyst in the presence of hydrogen. J. Appl. Polym. Sci..

[4] Ratanasak M., Parasuk V. (2016). Understanding the roles of novel electron donors in Ziegler-Natta catalyzed propylene polymerization. RSC. Adv..

[5] Kang J., Cao Y., Li H.L., Li J.P., Chen S.H., Yang F., Xiang M. (2012). Influence of the stereo-defect distribution on the crystallization behavior of Ziegler-Natta isotactic polypropylene. J. Polym. Res..

[6] Xu J.T., Feng L.X., Yang S.L. (1997). Formation mechanism of stereoblocks in polypropylene produced by supported Ziegler-Natta catalysts. Macromolecules.

[7] Credendino R., Liguori D., Morini G., Cavallo L. (2014). Investigating phthalate and 1,3-diether coverage and dynamics on the (104) and (110) surfaces of MgCl_2_-supported iegler-Natta catalysts. J. Phys. Chem. C.

[8] Bazhenov A., Linnolahti M., Pakkanen T.A., Denifl P., Leinonen T. (2014). Modeling the stabilization of surface defects by donors in Ziegler-Natta catalyst support. J. Phys. Chem. C.

[9] Potapov A.G., Politanskaya L.V. (2013). The study of the adsorption of 1,3-diethers on the MgCl_2_ surface. J. Mol. Catal. A Chem..

[10] Correa A., Piemontesi F., Morini G., Cavallo L. (2007). Key elements in the structure and function relationship of the MgCl_2_/TiCl_4_/lewis base Ziegler-Natta catalytic system. Macromolecules.

[11] Song B.G., Ihm S.K. (2014). The role of two different internal donors (Phthalate and 1,3-Diether) on the formation of surface structure in MgCl_2_-supported Ziegler-Natta catalysts and their catalytic performance of propylene polymerization. Appl. Polym. Sci..

[12] Pirinen S., Pakkanen T.T. (2015). Polyethers as potential electron donors for Ziegler-Natta ethylene polymerization catalysts. J. Mol. Catal. A Chem..

[13] Mignogna A., Balboni D., Cristofori A., Guidotti S., Morini G., Joachim T.M.P. (2014). Catalyst Components for the Polymerization of Olefins. U.S. Patent.

[14] Chen L.F., Gonzalez K. (2013). Catalyst Composition with Alkoxyalkyl Ester Internal Electron Donor and Polymer from Same. U.S. Patent.

[15] Chen B., Zhang Q.F., Zhao L.P., Zhang X.Q., Zhang H.X. (2013). Preparation and properties of isotactic polypropylene obtained from MgCl_2_-supported TiCl_4_ catalyst bearing bifunctional internal donor. Polym. Bull..

[16] Li H.Y., Zhou Q., Li Q., Zhang L.Y., Hu Y.L. (2014). Polypropylene Catalyst Containing an External Electron Donor of Carbonate. CN. Patent.

[17] Von der Assen N., Bardow A. (2014). Life cycle assessment of polyols for polyurethane production using CO_2_ as feedstock: Insights from an industrial case study. Green Chem..

[18] Ma K., Bai Q., Zhang L., Liu B.Y. (2016). Synthesis of flame-retarding oligo (carbonate-ether) diols via double metal cyanide complex-catalyzed copolymerization of PO and CO_2_ using bisphenol A as a chain transfer agent. RSC Adv..

[19] Gao Y.G., Qin Y.S., Zhao X.J., Wang F.S., Wang X.H. (2012). Selective synthesis of oligo (carbonate-ether) diols from copolymerization of CO_2_ and propylene oxide under zinc-cobalt double metal cyanide complex. J. Polym. Res..

[20] Makwana U.C., Singala K.J., Patankar R.B., Singh S.C., Gupta V.K. (2012). Propylene polymerization using supported Ziegler-Natta catalyst systems with mixed donors. J. Appl. Polym. Sci..

[21] Wen X., Ji J.M., Yi Q.F., Niu H., Dong J.Y. (2010). Magnesium chloride supported Ziegler-Natta catalysts containing succinate internal electron donors for the polymerization of propylene. J. Appl. Polym. Sci..

[22] Salakhov I.I., Batyrshin A.Z., Sergeev S.A., Bukatov G.D., Barabanov A.A., Mats’ko M.A., Sakhabutdinov A.G., Zakharov V.A. (2016). Effect of titanium-magnesium catalyst morphology on the properties of polypropylene upon propylene polymerization in a liquid monomer. Catal. Ind..

[23] Kaur S., Bantu B., Singu G., kumar N., Kapur G.S., Basu B. (2016). Particle Size Distribution Control through Internal Donor in Ziegler-Natta Catalyst. U.S. Patent.

[24] Fregonese D., Glisenti A., Mortara S., Rizzi G.A., Tondello E., Bresadola S. (2002). MgCl_2_/TiCl_4_/AlEt_3_ catalytic system for olefin polymerisation: A XPS study. J. Mol. Catal. A Chem..

[25] Xiao S.J., Cai S.M., Chen Z.P., Liu H.Q. (1986). The structural study of supported Ziegler-Natta catalysts for the polymerization of olefin. Stud. Surf. Sci. Catal..

[26] Mousty-Desbuquoit C., Riga J., Verbist J.J. (1983). Solid state effects in the electronic structure of TiCl_4_ studied by XPS. J. Chem. Phys..

[27] Mori H., Hasebe K., Terano M. (1999). Variation in oxidation state of titanium species on MgCl_2_-supported Ziegler catalyst and its correlation with kinetic behavior for propylene polymerization. Polymer.

[28] Mori H., Hasebe K., Terano M. (1999). XPS study of the interaction of titanium species with internal electron donors on MgCl_2_-supported Ziegler catalysts. J. Mol. Catal. A Chem..

[29] Korányi T., Magni I.E., Somorjai G.A. (1999). Surface science approach to the preparation and characterization of model Ziegler-Natta heterogeneous polymerization catalysts. Top. Catal..

[30] Ren H.G., Yang M., Zhang B.J., Ren X.F., Liu B.Y., Wang Y.J., Kim II. (2012). Isospecific polymerizations of 1-butene catalyzed by MgCl_2_/TiCl_4_/internal donor-AlR_3_/external donor system. Macromol. Res..

[31] Stukalov D.V., Zakharov V.A. (2009). Active site formation in MgCl_2_-supported Ziegler-Natta catalysts. A density functional theory study. J. Phys. Chem. C.

[32] Koivumäki J., Seppälä J.V., Kuutti L. (1992). Effect of the cocatalyst on the copolymerization of ethylene and propylene with high activity Ziegler-Natta catalyst. Polym. Bull..

[33] Dang X.F., Li Q., Li H.Y., Yang Y., Zhang L.Y., Hu Y.L. (2014). Ziegler-Natta catalysts with novel internal electron donors for propylene polymerization. J. Polym. Res..

[34] Zhang H.X., Lee L.Y., Park J.R., Lee D.H., Yoon K.B. (2011). Control of molecular weight distribution for polypropylene obtained by a commercial Ziegler-Natta catalyst: Effect of a cocatalyst and hydrogen. J. Appl. Polym. Sci..

[35] Chien J.C.W., Hu Y.L. (1988). Superactive and stereospecific catalysts. II. Kinetics of propylene polymerizations. J. Polym. Sci. Part A Polym. Chem..

[36] Chadwick J.C., van Kessel G.M.M., Sudmeijer O. (1995). Regio-and stereospecificity in propene polymerization with MgCl_2_-supported Ziegler-Natta catalysts: Effects of hydrogen and the external donor. Macromol. Chem. Phys..

[37] Chadwick J.C., Morini G., Balbontin G., Mingozzi I., Albizzati E., Sudmeijer O. (1997). Propene polymerization with MgCl_2_-supported catalysts: Effects of using a diether as external donor. Macromol. Chem. Phys..

